# Squamous Cell Carcinoma of the Oral Cavity, Oropharynx, and Larynx: A Scoping Review of Treatment Guidelines Worldwide

**DOI:** 10.3390/cancers15174405

**Published:** 2023-09-03

**Authors:** Lady Paola Aristizabal Arboleda, Genival Barbosa de Carvalho, Alan Roger Santos-Silva, Gisele Aparecida Fernandes, Jose Guilherme Vartanian, David I. Conway, Shama Virani, Paul Brennan, Luiz Paulo Kowalski, Maria Paula Curado

**Affiliations:** 1 Graduate Program of A.C.Camargo Cancer Center, Sao Paulo 01508-020, Brazil; paola.arboleda@accamargo.org.br; 2 Department of Head and Neck Surgery and Otorhinolaryngology, A.C.Camargo Cancer Center, Sao Paulo 01508-020, Brazil; 3 Oral Diagnosis Department, Piracicaba Dental School, University of Campinas, Piracicaba 13414-903, Brazil; 4 Group of Epidemiology and Statistics on Cancer, A.C.Camargo Cancer Center, Rua Tagua, 440, Liberdade, Sao Paulo CEP 01508-020, Brazil; 5 School of Medicine, Dentistry and Nursing, University of Glasgow, Glasgow G2 3JZ, UK; 6 Genomic Epidemiology Group, International Agency for Research on Cancer (IARC/WHO), G2 3JZ Lyon, France; 7 Department of Head and Neck Surgery, University of Sao Paulo Medical School, Sao Paulo 05403-000, Brazil

**Keywords:** cancer treatment, guidelines, head and neck cancer, larynx cancer, oral cancer, oropharynx cancer, scoping review

## Abstract

**Simple Summary:**

Treatment recommendations for head and neck cancer need to be disseminated worldwide becoming available through societies/authors scientific reports and websites with warning updates. This scoping review identifies and compares the worldwide clinical practice guidelines for treating oral, oropharynx, and larynx cancer. We verified the absence of guidelines in Latin American and Oceanian countries, as well as the inequalities between countries/continents, with a similar pattern of recommendations among low-income countries and in developed ones. Recommendations for surgery, radiotherapy, and chemotherapy may differ according to country/institution access and resources

**Abstract:**

Head and neck cancer (HNC) treatments have been based on single or multimodal therapies with surgery, radiotherapy (RT), chemotherapy, and immunotherapy. However, treatment recommendations among countries may differ due to technological/human resources and usual local practices. This scoping review aims to identify, compare, and map the clinical practice guidelines (CPGs) for treating squamous cell carcinoma (SCC) of the oral cavity, oropharynx, and larynx worldwide. A search strategy on global CPGs for HNC was performed by using five electronic databases and grey literature. CPGs were selected for inclusion using EndNote-20 and Rayyan online software. No language or publication date restrictions were applied. The results were analyzed descriptively considering the most updated CPG version. In total, 25 CPGs covering the head and neck region (10), the larynx (7), the oral cavity (5), and the oropharynx (3), were found in 13 geographical regions, and 19 were developed by medical societies from 1996 to 2023. Surgery and RT remain the main modalities for early-stage HNC, with surgery preferred in low-resource countries, and RT in selected cases, especially in the larynx/oropharynx aiming to achieve a cure with organ preservation. Human papillomavirus infection for oropharyngeal SCC is not tested in some Asian countries and there is still no consensus to treat p16-positive cases differently from p16-negative. Recommendations for larynx preservation vary according to facilities in each country, however, individualized choice is emphasized. Inequality across countries/continents is evident, with a similar pattern of recommendations among developed as well as developing ones. No CPGs were found in Latin America as well as Oceania countries, where the incidence of HNC is high and limitations of access to treatment may be encountered.

## 1. Introduction

Squamous cell carcinoma (SCC) is the main head and neck cancer (HNC) worldwide, with an estimated 660,740 new cases in the oral cavity, larynx, and oropharynx in 2020 [1]. Tobacco use (various forms), betel quid/areca nut use, alcohol consumption, and human papillomavirus (HPV) infection are globally recognized risk factors for HNC, with some variations in frequency depending on the culture of each geographic region [2]. Treatment protocols including surgery, radiotherapy (RT), chemotherapy (ChT), and immunotherapy, are chosen based on the TNM staging system provided by the Union for International Cancer Control/American Joint Committee on Cancer (AJCC) [3], with single modality therapy for most early-stage HNC and multimodal approach for most advanced-stage HNC [4].

Nowadays, progress has been observed in HNC treatment techniques to reduce morbidities, increase the long-term life quality of patients, and improve oncological outcomes, with transoral laser surgery (TLS), transoral robotic surgery (TORS), intensity-modulated radiation therapy (IMRT), photodynamic therapy, and sonodynamic therapy being advanced modalities [5,6]. As different prognosis outcomes are noted according to HNC anatomical subtypes and clinicopathological features, e.g., HPV-related oropharyngeal cancer (OPC), protocols strategies, including RT dose/volume de-intensification, induction response-based therapy, transoral surgery, and de-intensification of adjuvant treatment, are being widely investigated in clinical trials [7].

The involvement of a multidisciplinary team with different specialties such as head and neck surgery, radiation oncology, medical oncology, plastic/reconstructive surgery, pathology, specialized nursing care, dentistry/prosthodontics, physical medicine/rehabilitation, speech and swallowing therapy, clinical nutrition, clinical social work, among others, are essential in planning the HNC treatment [4]. A list of clinical recommendations made by a panel of experts based on a summary of supporting scientific evidence is meant as a clinical practice guideline (CPG), a published statement that aims to assist healthcare professionals in decision making according to the optimization of patient care [8]. Generally, CPGs provide workup recommendations for diagnosis, staging, treatment, and follow-up based on levels of evidence (quality and quantity of relevant published studies), and grades of recommendation (strength of the recommendation, ranging from strongly recommended to never recommended) [9].

It is noteworthy that the opportunities for accessing the diagnosis/treatment of HNC may differ globally, predominantly in developing countries due to limited facilities in terms of technology, medical infrastructure, and human resources, as well as in developed countries where most patients without health insurance cannot afford to pay out of pocket for cancer HNC treatment [10,11]. In this line of reasoning, the main CPGs for HNC treatment are published by well-recognized societies located in high-income countries [4,12,13].

Since treatment recommendations worldwide need to be known and incorporated in clinical practice, particularly in countries with different incomes (low-middle), a scoping review was the preferred study design by the authors, rather than a systematic review, to map a comprehensive range of available literature on CPGs for HNC, providing an overview, as well as knowledge gaps, using the following questions: What are the CPGs currently available for treating oral cavity, oropharynx, and larynx SCC worldwide? Which are the countries, societies, agencies, or authors that provide these CPGs? Do recommendations differ between CPGs? The compilated information may be the baseline for global CPGs covering HNC treatment according to access, facilities, and resources in different geographic regions.

## 2. Methodology

The present scoping review was part of the HEADSpAcE study, an International Consortium that researches HNC in South America and Europe, coordinated by the International Agency for Research on Cancer (https://headspace.iarc.fr/ accessed on 31 January 2019). The methodology was based on the Preferred Reporting Items for Systematic Reviews and Meta-Analyses extension for Scoping Reviews (PRISMA-ScR) [14]. A protocol describing the research design was registered on the Open Science Framework (https://doi.org/10.17605/OSF.IO/EVFRU accessed on 9 June 2023).

### 2.1. Information Sources and Search

Medline/PubMed, Scopus, Embase, LILACS, Web of Science, and Google Scholar (a grey literature database) were searched for studies published until April 2023. Additionally, supplementary sources via organizations/agencies/societies and reference lists of selected papers were manually screened, looking for additional relevant studies. The search was conducted by combining two groups of keywords (HNC and CPGs), each of them containing their synonyms or related keywords, and combined with the Boolean operator “AND”. Supplementary Table S1 shows the search strategy used by each database.

### 2.2. Selection of Sources of Evidence

Once the search was completed, all citations were uploaded into EndNote 20 software (EndNote^®^, Clarivate Analytics, Philadelphia, PA, USA), and duplicate records were removed. The titles and abstracts of all studies identified in the electronic searches were read, excluding articles that did not meet the eligibility criteria using the online software Rayyan^®^ (Qatar Computing Research Institute, Doha, Qatar). The eligible articles were selected by reading the full text, and all the primary reasons for exclusions were registered.

### 2.3. Eligibility Criteria

The inclusion criteria were applied in accordance with the PCC (Population, Concept, and Context): Worldwide CPGs (context) with recommendations on treatment (concept) in patients diagnosed with SSC in the oral cavity, oropharynx, and larynx (population). No restrictions regarding geographic location, society, language, or year of publication were applied. When more than one CPG was produced by the same organization, the most up-to-date version was considered for the analysis.

The following exclusion criteria were applied: (1) CPGs without treatment recommendations (CPGs for screening, diagnosis, supportive care, referrals, among others); (2) CPGs focused entirely on unique techniques (surgical procedures, radiation techniques, and systemic therapies); (3) CPGs for treatment of HNC recurrences and metastases; (4) CPGs on HNC topographies other than the oral cavity, oropharynx, and larynx; (5) non-SCC CPGs; (6) non-CPGs study designs (clinical trials, cohort studies, case–control studies, cross-sectional studies, case-series, case reports, reviews, personal opinions, letters, posters, conference abstracts, laboratory research (both in vivo and in vitro), and book chapters); (7) full texts not available; and (8) outdated versions of CPGs published by the same societies.

### 2.4. Data Synthesis and Descriptive Analysis

From the included studies, a data sheet utilizing the Microsoft Excel software was created for the extraction of data regarding the CPGs’ characteristics (year of publication, authoring societies/organizations, country or region, topography covered by the CPGs), the reason for the exclusion criteria, and key recommendations stated. The results were analyzed descriptively.

## 3. Results

### 3.1. Selection of Sources of Evidence

**Figure 1:**
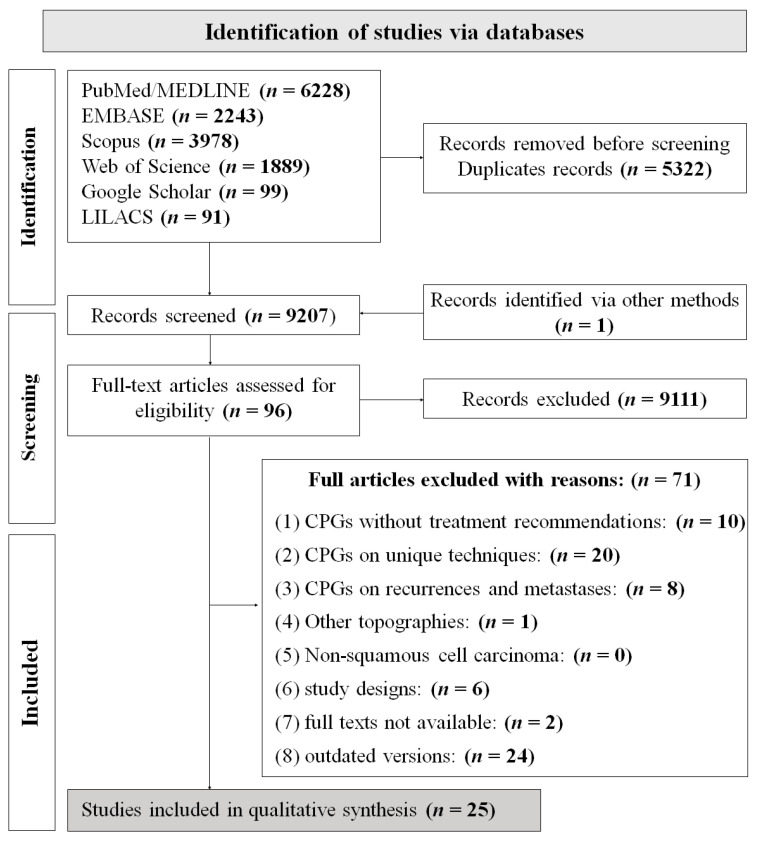
Flow diagram of literature search and selection criteria adapted from PRISMA 2020 [40].

The search resulted in 14,528 identified records, and 9206 records remained after duplicates were removed. One CPG was provided by the additional search [15]. A total of 9111 references were excluded during the initial screening of titles and abstracts and the remaining 96 studies moved to phase 2 of study selection. After full-text assessment, 25 studies were included in this scoping review (Figure 1), [13,15,16,17,18,19,20,21,22,23,24,25,26,27,28,29,30,31,32,33,34,35,36,37,38,39] and 71 studies were excluded (Supplementary Table S2).

### 3.2. Guideline Characteristics

**Figure 2:**
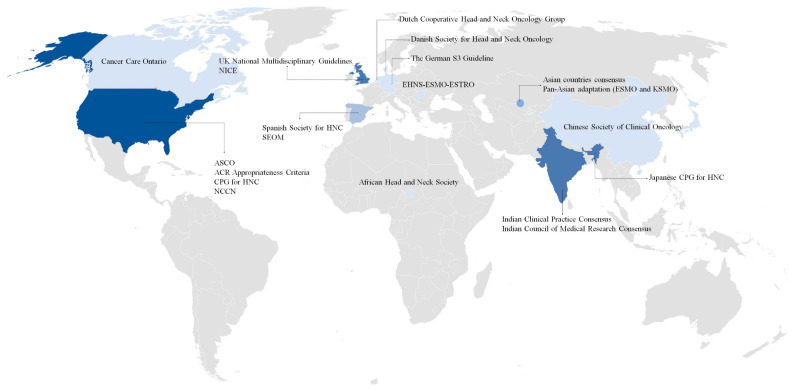
Societies producing treatment guidelines for head and neck cancer by country. Abbreviation: ACR, American College of Radiology; ASCO, American Society of Clinical Oncology; CPG, Clinical Practice Guidelines; EHNS, European Head and Neck Society; ESMO, European Society for Medical Oncology; ESTRO, European Society for Radiotherapy and Oncology; HNC, Head and neck cancer; KSMO, Korean Society of Medical Oncology; NCCN, National Comprehensive Cancer Network; NICE, National Institute for Health and Care Excellence; SEOM, Spanish Society of Medical Oncology; UK, United Kingdom.

A total of 25 CPGs were published between 1996 and 2023, 10 of them with algorithms guiding the recommendations [13,15,16,18,21,27,31,35,37,38]. CPGs with constant updates were observed in societies such as the Spanish Society of Medical Oncology (SEOM), American Society of Clinical Oncology (ASCO), European Society for Medical Oncology (ESMO), National Comprehensive Cancer Network (NCCN), and the American College of Radiology Appropriateness Criteria (ACR-AC). A total of 15 CPGs reported the TNM editions used for recommendations, 1 from the fifth edition (1997) [18], 6 from the seventh edition (2009) [22,23,25,28,30,32], and 8 from the eighth edition (2017) [13,15,16,21,27,31,35,38]. Three European CPGs used the Infectious Diseases Society of America-US Public Health Service Grading System for ranking strength of recommendations and quality of evidence score [13,27,31]. Regarding geographic areas, ten CPGs were from Europe [13,18,19,25,26,28,29,30,31,32,34], eight were from Asia [16,21,22,23,27,33,35,38], six CPGs from North America [15,17,24,36,37,39], and one from Africa [20]. Figure 2 represents the CPGs distributed by countries where the recommendations were performed, including the USA with the highest number of CPGs (4), followed by Spain, the United Kingdom, and India, with 2 for each. There were ten CPGs focused on HNC as a whole [13,15,16,22,27,29,31,32,33,34,37], seven were specific to the larynx [19,24,25,26,36,38,39], five for the oral cavity [18,20,21,23,28], and three for oropharynx [17,30,35]. The societies or professional organizations that produced CPGs for HNC treatment are shown per country and anatomical site in Table 1.

Regarding the recommendations that depended on the resources of each country, the following points were found: Since diagnostic imaging is limited in Africa, the CPG recommended its use preferably in invasive cases where management of the neck is required [20]. CPGs from Denmark published in 2006 reported limited access to CT and MRI [18]. HPV testing in OPC is not routinely included in CPGs in Asia and China [16,22]. CPGs from India recommended the use of conventional RT (2D/3D conformal therapy by cobalt 60 for external beam RT) [41], and surgery for oral cancers due to the limited number of facilities for brachytherapy [21]. Asia reported differences in drug availability for systemic therapy compared to Europe [13,27]. UK recognized that the most advanced treatments with the best evidence, are concentrated in the main centers of each country, making coverage difficult for the population that lives far away [32,34]. Table 2 presents a compilation of all CPG treatment recommendations for oral, oropharyngeal, and laryngeal SCC according to clinical stage, as well as the particularities found in terms of resources/limitations in some countries.

Treatment recommendations for oral cancer were provided by 15 CPGs, [13,15,16,18,20,21,22,23,27,28,29,31,32,33,34,37] with single modality treatment as the preferred option for early stages, with surgery being the main approach in all CPGs, and postoperative RT recommended for selected patients in the US, European, and Asian CPGs [13,27,37]. Regarding neck management, elective neck dissection (ipsilateral or bilateral) is indicated in most early-stage oral SCC cases; however, sentinel lymph node (SLN) biopsy has gained relevance, recommended by the UK, USA, Spain, and India [15,21,28,31,32,34]. Observation in clinically node-negative or, when the depth of invasion is 3 mm or less, was an option in CPGs from Africa and India [20,23]. Surgery remains the first line of treatment recommended in advanced stages, and probably the unique option in some African countries [20]. Multimodal therapy with postoperative RT or concomitant chemoradiotherapy (CCRT) in patients who are not candidates for or refuse radical surgery are recommendations in most countries [16,18,21,22,23,28,29,31,37]; however, clinical trials as a second treatment pathway for T1–3, N0–3; T4a, N0–3 are recommended in the USA [15]. There were CPGs advising the appropriate deep margin when the surgical modality is the choice, with 1 cm as the preferred free margin around the tumor in all dimensions [18,20,23,28]. Supplementary Table S3 displays the main recommendations for early and advanced oral SCC per country and society.

A total of 13 CPGs were found for OPC (Supplementary Table S4) [13,15,16,17,22,27,29,30,31,32,33,34,35,37]. Transoral/open resection of primary or RT were the main recommendations in the early stages, regardless of HPV status, except in Spain, because even acknowledging that there is no consensus, the panel recommended RT as the first treatment option in HPV-positive cases and surgery preferred in early HPV-negative OPC [29]. Surgery was preferred in most CPGs, with special surgical techniques such as TLS or TORS mentioned mostly by high-income countries [15,17,30,31,39]. Ipsilateral/bilateral neck dissection was recommended for all CPGs, and alternative options as prophylactic RT or SLN biopsy were from Spain [31]. CCRT is the main treatment modality as the first option recommended in advanced OPC; however, surgery is preferred in CPGs from China, Spain, and the USA [16,31,37]. Among cases treated with radical CCRT for primary, the neck should be evaluated by a PET-CT scan (positron emission tomography) after treatment, with a subsequent neck dissection if residual nodal disease is detected. When surgery is the primary option, postoperative RT alone may be indicated [30]. Clinical trials with de-intensification protocols for HPV-related OPC were mentioned in just a few studies [15,31].

Larynx cancer treatment has been widely studied, with a total of 17 CPGs found [13,16,19,22,24,25,26,27,29,31,32,33,34,36,37,38,39]. Both surgical interventions (endoscopic resection, partial laryngectomy, or open resection) and RT alone are treatment options in early-stage laryngeal cancer, with a preference for laser microsurgery [16,19,22,32,34,38], and RT for supraglottic cancer in some Asian CPGs [22,33]. For patients with locally advanced laryngeal SCC (stages III–IVA), modalities such as surgery, RT alone, CCRT, and induction chemotherapy (ICT) are options. Most guidelines showed CCRT as the preferred modality in terms of locoregional tumor control and overall survival when functional laryngeal preservation is feasible [16,19]. Elective neck dissection is the preferred modality in early-stage laryngeal SCC, except when the tumor is located in the glottic area [15,16,19,24,25,26,31,33]. For advanced stages, unilateral/bilateral elective neck dissection or therapeutic neck dissection is indicated [19]. PET-CT scan is a recent imaging recommendation for the evaluation of regional nodes after treatment, as well as swallowing function (Supplementary Table S5) [24,25].

## 4. Discussion

This is the first scoping review that investigates the worldwide guidelines for HNC treatment. Throughout this review, we provide an overview of the different approach recommendations according to geographic regions, as well as the lack of information that still exists, especially in low-income countries. Differences found among CPGs suggest inequity of health system conditions represented by the availability of resources such as imaging, health care professionals, technological advances in curative treatments, postoperative support, and the possibility of reconstruction and functional rehabilitation (Table 2).

Noteworthy, almost all geographic regions were found (Figure 2), with most CPGs published by societies representing countries such as the USA, India, the UK, and Spain [13,15,21,22,23,24,29,31,35,36,37,38]. Oceania and Latin American countries were the only regions without CPGs within this study, probably due to private CPGs by institutions with no access to the public using scientific databases, or the absence of national societies that publish CPGs by the general conditions of the countries. The increased publication of the guidelines between 2016 and 2020 occurred at the same time as the transition from the new 8th edition of the TNM implemented in January 2018, with a new classification for p16-positive OPC (T4 and N category reclassified), the extent of depth invasion in lip and oral cavity (T1–T3), and extranodal extension for non-HPV related tumors (N3 category subdivided into N3a and N3b) [42].

Recommendations aligned with the socioeconomic conditions of the region were reported only in an African CPG; however, it was focused on oral cancer, and surgical recommendations are preferred both in early and advanced stages, just with challenges when adjuvant therapy (ChT and RT) is needed and eventually not available [20]. The context and recommendations for treatment in the larynx and oropharynx cannot be applied in low-resource countries, since these types of cancer have higher recommendations for expensive surgical technologies (laser microsurgery or robotic), RT, and ChT, even in the early stages. Resource limitations are the most important reasons for different recommendations among countries. For example, the limited number of facilities for brachytherapy in India, as well as the use of conventional RT (2D/3D conformal therapy by cobalt 60 for external beam RT), which is still recommended, as it assists in wider accessibility of the treatment for a larger number of Indian patients [21,38,41]. In this regard, innovative treatment modalities such as TORS, TLM, and IMRT, which require infrastructure with the availability of specific devices, are only mentioned in some CPGs, mainly in those high-income countries [15,16,17,25,26,30,31,39]. Another important issue reported by a UK CPG is that some types of treatment, especially those that are more advanced and with better evidence of oncological results, are concentrated in the main centers of each country, making coverage difficult for the population that lives far away [32,34]. Even with technological and facilities resources, the cost of treatment represents a barrier to advanced treatment access [36].

Regarding oral SCC, the depth of invasion is a new pathological feature that must be reported to classify the clinicopathologic stage as well as to plan the treatment protocol [31]. However, the detection of the depth of invasion in Chinese patients with oral cancer is not performed as a routine [16]. Lip cancer was excluded in some CPGs and included in others as a whole in the oral cavity with the same treatment recommendations. Nevertheless, Asian CPGs reported that excision may not be the preferred initial treatment due to the unavailability of specialist reconstructive surgeons and infrastructure, which may reflect negative functional and aesthetic outcomes [22].

When it comes to the management of the neck in early-stage oral cancer, selective neck dissection is recommended in almost all CPGs and rarely prophylactic RT [31]. Various CPGs recommend SLN biopsy as it has gained evidence in terms of decreasing morbidity when compared to elective neck dissection [21,31,32,34]. However, as SLN biopsy is a relatively new approach, it requires a trained surgeon, and not all treatment center counts on human resources specialized in this surgical technique [32,34].

OPC has well-recognized clinicopathological and survival differences according to the HPV status, and even though there is still no consensus that they should be treated differently as the prognosis seems to be treatment-independent [17,30], the HPV-p16 identification is recommended. In general, all OPC should be tested using p16 immunohistochemistry and/or DNA or RNA in situ hybridization [35]. However, there are still countries that do not include routine HPV testing in oropharyngeal tumors as in some Asian and Chinese regions due to the lack of feasibility outside the clinical trial setting, and unclear HPV incidence rates in OPC [16,22]. Although several clinical trials on HNC treatment continue to be researched, OPC is of special interest to differentiate the approaches between positive and negative HPV cases. De-intensification or de-escalation of treatment is not recommended as a curative treatment in HPV-positive cases; however, it is mentioned in some guidelines as an option, only in clinical trial settings due to the lack of strong evidence that still exists [15,17,30,31,34]. Protocol approaches based on RT/systemic therapy de-escalation may be a treatment opportunity for HPV-related OPC patients, who have different clinicopathological behavior and much better prognosis than those with tobacco/alcohol disease. Thus, recent advances in the management of OPC via clinical trials are being performed to reduce toxicity, obtain the same prognostic response, and improve functional outcomes [7].

Generally, both surgery and RT are equally recommended in early-stage oropharynx and larynx cancer in terms of survival outcomes [16,24,25,39]. The difference in recommendations between these two modalities in most cases depends on the availability of RT, therefore, in countries where RT is a barrier or may have problems regarding waiting time for treatment, surgery is preferred [20,33]. On the other hand, there were countries that recommended advanced surgical techniques such as TLS or TORS and, despite positive oncological outcomes observed with these innovative techniques, not all institutions have facilities and experienced professionals to perform these surgical approaches.

As observed from all CPGs, except in a CPG published in 1996 where the larynx-preservation approach was not discussed as an option [37], functional organ preservation in advanced laryngeal cancer should be the main choice with CCRT [24,25]; however, it depends on several factors (patient factors, local expertise, and the availability of appropriate support and rehabilitative services). In the UK, partial laryngeal surgery is subject to the availability of expertise and multidisciplinary rehabilitation services since they do not have enough experience in these surgical techniques [25]. Although ICT appears as an option in advanced laryngeal cancer in organ-preservation cases, there were guidelines indicating insufficient evidence of survival or improved outcomes when ICT is applied before organ-preservation surgery or before concurrent treatment with altered fractionation RT [24,25]. One of the most decisive approaches to laryngeal cancer must be the high cost of organ preservation with ChT and/or RT, as well as the availability of facilities related to the treatment of acute and late toxicities offered by CCRT. It would be a major problem in countries where patients have limited access to medical care or due to ethnic disparities in health care [24,43]. In fact, mortality rate differences among socioeconomic groups have been demonstrated in laryngeal SCC, with higher mortality rates in patients with lower socioeconomic conditions [44].

Although the main objective of this study was primary treatment, adjuvant therapy recommendations varied among CPGs, adverse features, and the availability of proposed treatments. Perineural/vascular/lymphatic invasion, T3 or T4 primary, and neck positive (positive level IV or V nodes), are some of the features to consider adjuvant therapy, with RT alone, CCRT, ICT, and salvage neck dissection as an option according to the anatomical site and patients performance [13,15,27,31]. When ChT is chosen as part of the adjuvant protocol, cisplatin is the drug of choice; however, for unfit patients not candidates for platinum, cetuximab is another option, [13,27] except in Spain CPGs, since the panel does not recommend using agents such as cetuximab or carboplatin in the adjuvant setting due to lack of evidence [31].

Considering the functional and psychosocial impacts caused by HNC treatment, professional–patient communication is an important skill, as professionals should inform patients precisely about foreseeable functional sequelae that may affect them in different possible treatments and thus, be able to select, in mutual consensus, the best treatment option. Recommendations on topics that should be discussed with patients were included in some CPGs, with advantages of the proposed treatment, risks and associated complications after treatment, and impaired function according to each modality as the main points deciding treatment protocol [19,24,32,34].

Despite the attempt to cover worldwide CPG, the search strategy is beyond the scope of the CPG provided by specific institutions, making it necessary to create CPG by societies representing the country or continent as a whole. In addition, even performing a manual search of the literature, no access was allowed by some societies with archived CPG such as the Scottish Intercollegiate Guidelines Network and Cancer Care Ontario. Certain CPG characteristics such as lack of algorithms, recommendations without anatomical and staging specification (TNM), different criteria for assessing the level and quality of evidence, and a grade of recommendation, were some limitations of this scoping review since differences in this regard hamper comparations among CPGs. However, as strengths, we comprehensively describe the main characteristics of CPGs available in the literature for head and neck cancer treatment and, probably, the starting point for future CPGs with worldwide coverage according to each country’s reality.

## 5. Conclusions

Finally, from this overview of treatment recommendations, it is evident that there is still a lack of worldwide coverage for access to all HNC protocols and techniques. We observed a shortage of guidelines, especially in lower-middle-income countries such as those in Latin American and Oceania countries. Despite the differences and socioeconomic limitations of each country, a consensus should be sought among countries to unify some specific criteria for the treatment of HNC. However, while CPGs are needed to guide treatment choice, it is critical to recognize that individual factors, such as patient characteristics, comorbidities, preferences, and the healthcare system, may determine different treatment pathways.

## Figures and Tables

**Table 1:** Clinical practice guidelines description per countries, societies/organizations, and anatomical site.

Country/Continent	Guideline	Society/Organization	Anatomical Site Covered	Year of Publication
Africa	Guidelines for low-resource regions	African Head and Neck Society	Oral Cavity	2019
Asia	Consensus recommendations for management of HNC in Asian countries	The Asia Pacific HNC Expert Panel	Head and Neck	2013
	Pan-Asian Adaptation of the European Society for Medical Oncology	European Society for Medical Oncology and Korean Society of Medical Oncology	Head and Neck	2021
Canada	Clinical practice guideline	Cancer Care Ontario’s HNC disease site group	Larynx	2013
China	Diagnosis and treatment guidelines for HNC working group	Chinese Society of Clinical Oncology	Head and Neck	2019
Denmark	The Danish National Guidelines	Danish Society for Head and Neck Oncology	Oral Cavity	2006
Europe	EHNS-ESMO-ESTRO Clinical Practice Guidelines	The EHNS Executive Board, ESMO Guidelines Committee, and ESTRO Executive Board	Head and Neck	2020
Germany	The German S3 Guideline	German Cancer Society and German Cancer Aid	Larynx	2020
India	Indian Clinical Practice Consensus	Oral Cancer Task Force with a Multidisciplinary Expert Panel	Oropharynx	2020
			Oral Cavity	2020
			Larynx	2020
	Indian Council of Medical Research Consensus	Indian Council of Medical Research	Oral cavity	2015
Japan	Japanese Clinical Practice Guideline for HNC	Japan Society for Head and Neck Cancer	Head and Neck	2017
Spain	Spanish multidisciplinary consensus	Spanish Society for Head and Neck Cancer	Head and Neck	2017
	Spanish Society of Medical Oncology	Spanish Group for the Treatment of Head and Neck Tumors and SEOM	Head and Neck	2021
Netherlands	The Dutch National Guideline	Dutch Cooperative Head and Neck Oncology Group	Larynx	2002
UK	United Kingdom National Multidisciplinary Guidelines	Specialty associations involved in the care of HNC in the UK	Oropharynx	2016
			Oral cavity	2016
			Larynx	2016
	National Institute for Health and Care Excellence Guidelines for England and Wales	NICE Guideline Committee	Head and Neck	2016
USA	American Society of Clinical Oncology Clinical Practice Guideline	American Society of Clinical Oncology	Larynx	2018
	American College of Radiology Appropriateness Criteria	The ACR Expert Panel on Radiation Oncology—HNC	Oropharynx	2016
			Larynx	2014
	Clinical Practice Guidelines for HNC	H. Lee Moffitt Cancer Center and Research Institute-Clinical Practice Guidelines Committee for the Head and Neck Program	Head and Neck	1996
	National Comprehensive Cancer Network Guidelines	NCCN Head and Neck Cancers Panel Members	Head and Neck	2023

Abbreviations: HNC, head and neck cancer; NCCN, National Comprehensive Cancer Network; NICE, National Institute for Health and Care Excellence; SEOM, Spanish Society of Medical Oncology; EHNS, European Head and Neck Society; ESMO, European Society for Medical Oncology; ESTRO, European Society for Radiotherapy and Oncology.

**Table 2:** Treatment recommendations for oral, oropharyngeal, and laryngeal SCC according to clinical stage and resources/limitations by countries.

Cancer Type	Oral Cancer		Oropharyngeal Cancer		Laryngeal Cancer	
Clinical Stage	Early	Advance	Early	Advance	Early	Advance
Standard therapy recommendations *	Surgery (preferred) or RT Elective neck dissection (ipsilateral or bilateral)	Surgery (preferred),RT, or CCRT	Transoral/open resection or RT Ipsilateral/bilateral neck dissection	CCRT or surgery followed by postoperative RT/CRT or ICT	Surgery (endoscopic resection, partial laryngectomy, or open resection) or RT Elective neck dissection (except in the early-stage glottic cancer)	Surgery, RT, CCRT, and ICT Neck dissection
Recommendations according to limitations/resources of each country	⮚ Africa: challenges when CRT is needed, thus, surgery is eventually considered as a single option [20]. ⮚ Japan: limited number of brachytherapy facilities [33]. ⮚ India: surgery preferred (simplicity, low cost, minimal change in function, and repeatability) [23]. ⮚ Asia: unavailability of reconstructive surgeons and infrastructure [22]. ⮚ UK, USA, Spain, and India: SLN biopsy recommended [15,21,28,31,32,34]. ⮚ Africa and India: observation in clinically node-negative or, when the depth of invasion is 3 mm or less [20,23].		⮚ China, Asia: non-routine HPV-p16 testing [16,22]. ⮚ Spain: RT is preferred in HPV-positive cases and surgery in HPV-negative cases [29]. ⮚ Asia: RT is preferred in T1-2N0. Surgery reserved for patients with limited tonsil disease and good functional status [22]. ⮚ USA, UK, Spain: TLS and TORS are techniques recommended [15,17,30,31]. ⮚ USA, Spain: Clinical trials with deintensification protocols for HPV-related OPC [15,31].		⮚ USA: high cost of CRT versus laryngectomy [24]. ⮚ UK: preserve the organ at an advanced stage with CRT [25]. ⮚ Netherlands: surgery only for in situ cases and RT as conservative voice therapy [26]. ⮚ Canada, China, Germany, UK, India: preference for TLS, TORS, IMRT [16,19,32,34,38,39]. ⮚ UK: partial laryngeal surgery is subject to the availability of expertise and multidisciplinary rehabilitation services since they do not have enough experience in these surgical techniques [25].	

Abbreviation: CRT, chemoradiotherapy; CCRT, concomitant chemoradiotherapy; HPV, human papillomavirus; ICT, induction chemotherapy; IMRT, intensity modulated radiotherapy; OPC; oropharyngeal cancer; SLN, sentinel lymph node; TLS, transoral laser surgery; TORS, transoral robotic surgery; RT, radiotherapy. * Standard therapy recommendations according to a summary of all clinical practice guidelines included in this scoping review.

## Data Availability

Data availability are available on the Open Science Framework (https://doi.org/10.17605/OSF.IO/EVFRU accessed on 9 June 2023). If additional data is needed, the authors would make it available according to reasonable requests.

## Supplementary Materials

The following supporting information can be downloaded at: https://www.mdpi.com/article/10.3390/cancers15174405/s1, Table S1: Search strategy used per database; Table S2: Articles excluded and the reasons for exclusion (*n* = 71); Table S3: Treatment recommendation for oral cavity per society according to clinical stages; Table S4: Treatment recommendation for oropharyngeal cancer per society according to clinical stages; Table S5: Treatment recommendation for larynx cancer per society according to clinical stages.
