# Supplementary material for: Squamous Cell Carcinoma of the Oral Cavity, Oropharynx, and Larynx: A Scoping Review of Treatment Guidelines Worldwide
Source: Cancers (Basel). 2023 Sep 3;15(17):4405. doi: 10.3390/cancers15174405 (PMC10486835; doi:10.3390/cancers15174405)
Supplement: Supplementary file 1 [file cancers-15-04405-s001.zip › cancers-2568544-supplementary.pdf]

Supplementary material

Table S1. Search strategy used per database

DATABASE	SEARCH STRATEGY Search date (April 12, 2023)	RESULTS
**PUPMED**	("Head and Neck Neoplasms"[Mesh] OR "Head and Neck Neoplasm*" OR "head and neck cancer" OR "head and neck malignant tumo*" OR "head and neck malignancies" OR "cancer of the head and neck" OR "Head and neck Carcinoma*" OR "Cancer of Mouth" OR "Mouth Cancer*" OR "Oral Cancer*" OR "Cancer of the Mouth" OR "Mouth Cancer" OR "Cancer of Lip" OR "Lip Cancer*" OR "Cancer of the Lip" OR "Jaw Cancer*" OR "Cancer of the Jaw" OR "Cancer of Tongue" OR "Tongue Cancer*" OR "Cancer of the Tongue" OR "Cancer of Larynx" OR "Larynx Cancer*" OR "Laryngeal Cancer" OR "Cancer of the Larynx" OR "Oropharyngeal Cancer*" OR "Cancer of the Oropharynx" OR "Cancer of Tonsil" OR "Tonsil Cancer*" OR "Cancer of the Tonsil" OR "Tonsillar Cancer*") AND ("Guidelines as Topic"[Mesh] OR "Practice Guidelines as Topic"[Mesh] OR "Guideline" [Publication Type] OR "Health Planning Guidelines"[Mesh] OR Guideline OR Guidelines OR "Planning Guideline*" OR "Guidelines for Health Planning")	**6,228**
**Scopus**	TITLE-ABS-KEY("Head and Neck Neoplasm*" OR "head and neck cancer" OR "head and neck malignant tumo*" OR "head and neck malignancies" OR "cancer of the head and neck" OR "Head and neck Carcinoma*" OR "Cancer of Mouth" OR "Mouth Cancer*" OR "Oral Cancer*" OR "Cancer of the Mouth" OR "Mouth Cancer" OR "Cancer of Lip" OR "Lip Cancer*" OR "Cancer of the Lip" OR "Jaw Cancer*" OR "Cancer of the Jaw" OR "Cancer of Tongue" OR "Tongue Cancer*" OR "Cancer of the Tongue" OR "Cancer of Larynx" OR "Larynx Cancer*" OR "Laryngeal Cancer" OR "Cancer of the Larynx" OR "Oropharyngeal Cancer*" OR "Cancer of the Oropharynx" OR "Cancer of Tonsil" OR "Tonsil Cancer*" OR "Cancer of the Tonsil" OR "Tonsillar Cancer*") AND TITLE-ABS-KEY("Guidelines as Topic" OR "Practice Guidelines as Topic" OR "Health Planning Guidelines" OR Guideline OR Guidelines OR "Planning Guideline*" OR "Guidelines for Health Planning")	**3,978**
**EMBASE**	('head and neck neoplasm*' OR 'head and neck cancer'/de OR 'head and neck malignant tumo*' OR 'head and neck malignancies' OR 'cancer of the head and neck' OR 'head and neck carcinoma*' OR 'cancer of mouth' OR 'mouth cancer*' OR 'oral cancer*' OR 'cancer of the mouth' OR 'mouth cancer'/de OR 'cancer of lip' OR 'lip cancer*' OR 'cancer of the lip' OR 'jaw cancer*' OR 'cancer of the jaw' OR 'cancer of tongue' OR 'tongue cancer*' OR 'cancer of the tongue' OR 'cancer of larynx' OR 'larynx cancer*' OR 'laryngeal cancer'/de OR 'cancer of the larynx' OR 'oropharyngeal cancer*' OR 'cancer of the oropharynx' OR 'cancer of tonsil' OR 'tonsil cancer*' OR 'cancer of the tonsil' OR 'tonsillar cancer*') AND ('guidelines as topic'/de OR 'practice guidelines as topic'/de OR 'health planning guidelines'/de OR 'guideline'/de OR 'guidelines'/de OR 'planning guideline*' OR 'guidelines for health planning')	**2,243**
**Web of Science**	("Head and Neck Neoplasm*" OR "head and neck cancer" OR "head and neck malignant tumo*" OR "head and neck malignancies" OR "cancer of the head and neck" OR "Head and neck Carcinoma*" OR "Cancer of Mouth" OR "Mouth Cancer*" OR "Oral Cancer*" OR "Cancer of the Mouth" OR "Mouth Cancer" OR "Cancer of Lip" OR "Lip Cancer*" OR "Cancer of the Lip" OR "Jaw Cancer*" OR "Cancer of the Jaw" OR "Cancer of Tongue" OR "Tongue Cancer*" OR "Cancer of the Tongue" OR "Cancer of Larynx" OR "Larynx Cancer*" OR "Laryngeal Cancer" OR "Cancer of the Larynx" OR "Oropharyngeal Cancer*" OR "Cancer of the Oropharynx" OR "Cancer of Tonsil" OR "Tonsil Cancer*" OR "Cancer of the Tonsil" OR "Tonsillar Cancer*") AND ("Guidelines as Topic" OR "Practice Guidelines as Topic" OR "Health Planning Guidelines" OR Guideline OR Guidelines OR "Planning Guideline*" OR "Guidelines for Health Planning")	**1,889**
**LILACS**	(("cancer oral" OR "oral cancer" OR "câncer oral" OR "oropharyngeal cancer" OR "cancer de orofaringe" OR "oropharynx cancer" OR "laryngeal cancer" OR "larynx cancer" OR "cancer de laringe")) AND ((guidelines OR diretrizes OR directrices))	**91**
**Google Scholar**	("oral cancer" OR "oropharyngeal cancer" OR "oropharynx cancer" OR "laryngeal cancer" OR "larynx cancer") AND (guidelines)	**99**
**Total**		**14,528**

Table S2. Articles excluded and the reasons for exclusion (n= 71)

Reasons for Exclusion*	Reference
1	de Monès, E., Vergez, S., Barry, B., Righini, C., Rolland, F., Raoul, G., Langeard, M., Chassagne, J. F., Badoual, C., Morinière, S., & de Raucourt, D. (2013). Initial staging for squamous cell carcinoma of the mouth, larynx and pharynx (except nasopharynx). Part 3: general assessment. 2012 SFORL recommendations. Eur Ann Otorhinolaryngol Head Neck Dis, 130(3), 165-172. https://doi.org/10.1016/j.anorl.2012.09.002
1	Vergez, S., Morinière, S., Dubrulle, F., Salaun, P. Y., De Monès, E., Bertolus, C., Temam, S., Chevalier, D., Lagarde, F., Schultz, P., Ferrié, J. C., Badoual, C., Lapeyre, M., Righini, C., Barry, B., Tronche, S., & De Raucourt, D. (2013). Initial staging of squamous cell carcinoma of the oral cavity, larynx and pharynx (excluding nasopharynx). Part I: Locoregional extension assessment: 2012 SFORL guidelines. Eur Ann Otorhinolaryngol Head Neck Dis, 130(1), 39-45. https://doi.org/10.1016/j.anorl.2012.09.004
1	Clinical Governance, R., & Development Unit, D. o. H. S. U. o. L. (2005). National Institute for Health and Clinical Excellence: Guidance. In Referral Guidelines for Suspected Cancer in Adults and Children. Royal College of General Practitioners (UK)
1	Irish, J., Kim, J., Waldron, J., Wei, A. C., Winquist, E., Yoo, J., Boasie, A., Brouwers, M., Meertens, E., McNair, S., & Walker-Dilks, C. (2020). Organizational guidance for the care of patients with head-and-neck cancer in Ontario. Curr Oncol, 27(2), e115-e122. https://doi.org/10.3747/co.27.5873
1	Robson, A., Sturman, J., Williamson, P., Conboy, P., Penney, S., & Wood, H. (2016). Pre-treatment clinical assessment in head and neck cancer: United Kingdom National Multidisciplinary Guidelines. J Laryngol Otol, 130(S2), S13-s22. https://doi.org/10.1017/s0022215116000372
1	Cuny, F., Babin, E., Lacau-Saint-Guily, J., Baujat, B., Bensadoun, R., Bozec, A., Chevalier, D., Choussy, O., Deneuve, S., Fakhry, N., Guigay, J., Makeieff, M., Merol, J. C., Mouawad, F., Pavillet, J., Rebiere, C., Righini, C., Sostras, M. C., Tournaille, M., & Vergez, S. (2015). French Society of ENT (SFORL) guidelines for care pathway organization in head and neck oncology (short version). Early management of head and neck cancer. Eur Ann Otorhinolaryngol Head Neck Dis, 132(4), 205-208. https://doi.org/10.1016/j.anorl.2015.06.007
1	Grégoire, V., Ang, K., Budach, W., Grau, C., Hamoir, M., Langendijk, J. A., Lee, A., Le, Q. T., Maingon, P., Nutting, C., O'Sullivan, B., Porceddu, S. V., & Lengele, B. (2014). Delineation of the neck node levels for head and neck tumors: a 2013 update. DAHANCA, EORTC, HKNPCSG, NCIC CTG, NCRI, RTOG, TROG consensus guidelines. Radiother Oncol, 110(1), 172-181. https://doi.org/10.1016/j.radonc.2013.10.010
1	Wee, J. T., Anderson, B. O., Corry, J., D'Cruz, A., Soo, K. C., Qian, C. N., Chua, D. T., Hicks, R. J., Goh, C. H., Khoo, J. B., Ong, S. C., Forastiere, A. A., & Chan, A. T. (2009). Management of the neck after chemoradiotherapy for head and neck cancers in Asia: consensus statement from the Asian Oncology Summit 2009. Lancet Oncol, 10(11), 1086-1092. https://doi.org/10.1016/s1470-2045(09)70266-9
1	O'Connell, D. A., Seikaly, H., Isaac, A., Pyne, J., Hart, R. D., Goldstein, D., & Yoo, J. (2020). Recommendations from the Canadian Association of Head and Neck Surgical Oncology for the Management of Head and Neck Cancers during the COVID-19 pandemic. J Otolaryngol Head Neck Surg, 49(1), 53. https://doi.org/10.1186/s40463-020-00448-z
1	Mehanna, H., Hardman, J. C., Shenson, J. A., Abou-Foul, A. K., Topf, M. C., AlFalasi, M., Chan, J. Y. K., Chaturvedi, P., Chow, V. L. Y., Dietz, A., Fagan, J. J., Godballe, C., Golusiński, W., Homma, A., Hosal, S., Iyer, N. G., Kerawala, C., Koh, Y. W., Konney, A., . . . Holsinger, F. C. (2020). Recommendations for head and neck surgical oncology practice in a setting of acute severe resource constraint during the COVID-19 pandemic: an international consensus. Lancet Oncol, 21(7), e350-e359. https://doi.org/10.1016/s1470-2045(20)30334-x
2	Yeung, A. R., Garg, M. K., Lawson, J., McDonald, M. W., Quon, H., Ridge, J. A., Saba, N., Salama, J. K., Smith, R. V., Yom, S. S., & Beitler, J. J. (2012). ACR Appropriateness Criteria® ipsilateral radiation for squamous cell carcinoma of the tonsil. Head Neck, 34(5), 613-616. https://doi.org/10.1002/hed.21993
2	Porras Alonso, E., Vilaseca González, I., García Teno, M., Barberá Durbán, R., Viscasillas Pallàs, G., Sancho Mestre, M., Rebollo Otal, J., Menoyo Bueno, A., & Díaz de Cerio Canduela, P. (2020). Early glottic tumours with anterior commissure involvement. Literature review and consensus document. Head and Neck and Skull Base Commission, SEORL-CCC. Acta Otorrinolaringol Esp (Engl Ed), 71 Suppl 1, 1-20. https://doi.org/10.1016/j.otorri.2020.02.008 (Tumores glóticos precoces con afectación de la comisura anterior. Revisión bibliográfica y documento de consenso. Comisión de cabeza y cuello y base de cráneo. SEORL-CCC.)

2	Grégoire, V., Evans, M., Le, Q. T., Bourhis, J., Budach, V., Chen, A., Eisbruch, A., Feng, M., Giralt, J., Gupta, T., Hamoir, M., Helito, J. K., Hu, C., Hunter, K., Johansen, J., Kaanders, J., Laskar, S. G., Lee, A., Maingon, P., . . . Grau, C. (2018). Delineation of the primary tumour Clinical Target Volumes (CTV-P) in laryngeal, hypopharyngeal, oropharyngeal and oral cavity squamous cell carcinoma: AIRO, CACA, DAHANCA, EORTC, GEORCC, GORTEC, HKNPCSG, HNCIG, IAG-KHT, LPRHHT, NCIC CTG, NCRI, NRG Oncology, PHNS, SBRT, SOMERA, SRO, SSHNO, TROG consensus guidelines. Radiother Oncol, 126(1), 3-24. https://doi.org/10.1016/j.radonc.2017.10.016
2	Jensen, K., Friborg, J., Hansen, C. R., Samsøe, E., Johansen, J., Andersen, M., Smulders, B., Andersen, E., Nielsen, M. S., Eriksen, J. G., Petersen, J. B. B., Elstrøm, U. V., Holm, A. I., Farhadi, M., Morthorst, M. H., Skyt, P. S., Overgaard, J., & Grau, C. (2020). The Danish Head and Neck Cancer Group (DAHANCA) 2020 radiotherapy guidelines. Radiother Oncol, 151, 149-151. https://doi.org/10.1016/j.radonc.2020.07.037
2	Bradley, P. J., MacKenzie, K., Wight, R., Pracy, P., & Paleri, V. (2009). Consensus statement on management in the UK: Transoral laser assisted microsurgical resection of early glottic cancer [Review]. Clinical Otolaryngology, 34(4), 367-373. https://doi.org/10.1111/j.1749-4486.2009.01944.x
2	Merlotti, A., Alterio, D., Vigna-Taglianti, R., Muraglia, A., Lastrucci, L., Manzo, R., Gambaro, G., Caspiani, O., Miccichè, F., Deodato, F., Pergolizzi, S., Franco, P., Corvò, R., Russi, E. G., & Sanguineti, G. (2014). Technical guidelines for head and neck cancer IMRT on behalf of the Italian association of radiation oncology - head and neck working group. Radiat Oncol, 9, 264. https://doi.org/10.1186/s13014-014-0264-9
2	Cohen, E. E. W., Bell, R. B., Bifulco, C. B., Burtness, B., Gillison, M. L., Harrington, K. J., Le, Q. T., Lee, N. Y., Leidner, R., Lewis, R. L., Licitra, L., Mehanna, H., Mel, L. K., Raben, A., Sikora, A. G., Uppaluri, R., Whitworth, F., Zandberg, D. P., & Ferris, R. L. (2019). The Society for Immunotherapy of Cancer consensus statement on immunotherapy for the treatment of squamous cell carcinoma of the head and neck (HNSCC). Journal for ImmunoTherapy of Cancer, 7, Article 184. https://doi.org/10.1186/s40425-019-0662-5
2	Cripps, C., Winquist, E., Devries, M. C., Stys-Norman, D., & Gilbert, R. (2010). Epidermal growth factor receptor targeted therapy in stages III and IV head and neck cancer. Curr Oncol, 17(3), 37-48. https://doi.org/10.3747/co.v17i3.520
2	Bhalavat, R., Budrukkar, A., Laskar, S. G., Sharma, D., Mukherji, A., Chandra, M., Mahantshetty, U., Pareek, V., Bauskar, P., & Saraf, S. (2020). Brachytherapy in head and neck malignancies: Indian Brachytherapy Society (IBS) recommendations and guidelines. J Contemp Brachytherapy, 12(5), 501-511. https://doi.org/10.5114/jcb.2020.100385
2	Lin, A., Chang, J. H. C., Grover, R. S., Hoebers, F. J. P., Parvathaneni, U., Patel, S. H., Thariat, J., Thomson, D. J., Langendijk, J. A., & Frank, S. J. (2021). PTCOG Head and Neck Subcommittee Consensus Guidelines on Particle Therapy for the Management of Head and Neck Tumors. Int J Part Ther, 8(1), 84-94. https://doi.org/10.14338/ijpt-20-00071.1
2	Alterio, D., Volpe, S., Bacigalupo, A., Bonomo, P., De Felice, F., Dionisi, F., D'Onofrio, I., D'Angelo, E., Di Rito, A., Fanetti, G., Franco, P., Maddalo, M., Merlotti, A., Micciché, F., Orlandi, E., Paiar, F., Ursino, S., Pepa, M., Corvò, R., . . . Musio, D. (2020). Head and neck radiotherapy amid the COVID-19 pandemic: practice recommendations of the Italian Association of Radiotherapy and Clinical Oncology (AIRO). Med Oncol, 37(10), 85. https://doi.org/10.1007/s12032-020-01409-2
2	Benasso, M., Bonomo, P., Buglione, M., Ghi, M. G., Licitra, L., Magrini, S. M., Merlano, M. C., Presutti, L., Ronzino, G., & Ferrari, D. (2020). Selection of systemic therapy in patients with locally advanced and recurrent/metastatic head and neck cancer: RAND-based expert opinion by an Italian multidisciplinary panel [Review]. Tumori, 106(3), 177-189. https://doi.org/10.1177/0300891619868289
2	Joo, Y. H., Cho, J. K., Koo, B. S., Kwon, M., Kwon, S. K., Kwon, S. Y., Kim, M. S., Kim, J. K., Kim, H., Nam, I., Roh, J. L., Park, Y. M., Park, I. S., Park, J. J., Shin, S. C., Ahn, S. H., Won, S., Ryu, C. H., Yoon, T. M., . . . Chung, P. S. (2019). Guidelines for the Surgical Management of Oral Cancer: Korean Society of Thyroid-Head and Neck Surgery. Clin Exp Otorhinolaryngol, 12(2), 107-144. https://doi.org/10.21053/ceo.2018.01816
2	Quon, H., Vapiwala, N., Forastiere, A., Kennedy, E. B., Adelstein, D. J., Boykin, H., Califano, J. A., Holsinger, F. C., Nussenbaum, B., Rosenthal, D. I., Siu, L. L., & Waldron, J. N. (2017). Radiation Therapy for Oropharyngeal Squamous Cell Carcinoma: American Society of Clinical Oncology Endorsement of the American Society for Radiation Oncology Evidence-Based Clinical Practice Guideline. J Clin Oncol, 35(36), 4078-4090. https://doi.org/10.1200/jco.2017.73.8633
2	Koyfman, S. A., Ismaila, N., Crook, D., D'Cruz, A., Rodriguez, C. P., Sher, D. J., Silbermins, D., Sturgis, E. M., Tsue, T. T., Weiss, J., Yom, S. S., & Christopher Holsinger, F. (2019). Management of the neck in squamous cell carcinoma of the oral cavity and oropharynx: ASCO clinical practice guideline [Review]. Journal of Clinical Oncology, 37(20), 1753-1774. https://doi.org/10.1200/JCO.18.01921
2	Winquist, E., Agbassi, C., Meyers, B. M., Yoo, J., & Chan, K. K. W. (2017). Systemic therapy in the curative treatment of head-and-neck squamous cell cancer: Cancer Care Ontario clinical practice guideline. Curr Oncol, 24(2), e157-e162. https://doi.org/10.3747/co.24.3489
2	Ahn, S. H., Hong, H. J., Kwon, S. Y., Kwon, K. H., Roh, J. L., Ryu, J., Park, J. H., Baek, S. K., Lee, G. H., Lee, S. Y., Lee, J. C., Chung, M. K., Joo, Y. H., Ji, Y. B., Hah, J. H., Kwon, M., Park, Y. M., Song, C. M., Shin, S. C., . . . Lee, K. D. (2017). Guidelines for the Surgical Management of Laryngeal Cancer: Korean Society of Thyroid-Head and Neck Surgery. Clin Exp Otorhinolaryngol, 10(1), 1-43. https://doi.org/10.21053/ceo.2016.01389
2	Homer, J. J., & Fardy, M. J. (2016). Surgery in head and neck cancer: United Kingdom National Multidisciplinary Guidelines. J Laryngol Otol, 130(S2), S68-s70. https://doi.org/10.1017/s0022215116000475
2	Kelly, C. G. (2016). Chemotherapy: United Kingdom National Multidisciplinary Guidelines. J Laryngol Otol, 130(S2), S71-s74. https://doi.org/10.1017/s0022215116000840
2	Salama, J. K., Haddad, R. I., Kies, M. S., Busse, P. M., Dong, L., Brizel, D. M., Eisbruch, A., Tishler, R. B., Trotti, A. M., & Garden, A. S. (2009). Clinical practice guidance for radiotherapy planning after induction chemotherapy in locoregionally advanced head-and-neck cancer. Int J Radiat Oncol Biol Phys, 75(3), 725-733. https://doi.org/10.1016/j.ijrobp.2008.11.059
3	de Monès, E., Bertolus, C., Salaun, P. Y., Dubrulle, F., Ferrié, J. C., Temam, S., Chevalier, D., Vergez, S., Lagarde, F., Schultz, P., Lapeyre, M., Barry, B., Tronche, S., de Raucourt, D., & Morinière, S. (2013). Initial staging of squamous cell carcinoma of the oral cavity, larynx and pharynx (excluding nasopharynx). Part 2: Remote extension assessment and exploration for secondary synchronous locations outside of the upper aerodigestive tract. 2012 SFORL guidelines. Eur Ann Otorhinolaryngol Head Neck Dis, 130(2), 107-112. https://doi.org/10.1016/j.anorl.2012.09.003
3	Halimi, C., Barry, B., De Raucourt, D., Choussy, O., Dessard-Diana, B., Hans, S., & Lafarge, D. (2015). Guidelines of the French Society of Otorhinolaryngology (SFORL), short version. Diagnosis of local recurrence and metachronous locations in head and neck oncology. Eur Ann Otorhinolaryngol Head Neck Dis, 132(5), 287-290. https://doi.org/10.1016/j.anorl.2015.07.002
3	McDonald, M. W., Lawson, J., Garg, M. K., Quon, H., Ridge, J. A., Saba, N., Salama, J. K., Smith, R. V., Yeung, A. R., Yom, S. S., & Beitler, J. J. (2011). ACR appropriateness criteria retreatment of recurrent head and neck cancer after prior definitive radiation expert panel on radiation oncology-head and neck cancer. Int J Radiat Oncol Biol Phys, 80(5), 1292-1298. https://doi.org/10.1016/j.ijrobp.2011.02.014
3	Paleri, V., Urbano, T. G., Mehanna, H., Repanos, C., Lancaster, J., Roques, T., Patel, M., & Sen, M. (2016). Management of neck metastases in head and neck cancer: United Kingdom National Multidisciplinary Guidelines. J Laryngol Otol, 130(S2), S161-s169. https://doi.org/10.1017/s002221511600058x
3	Mehanna, H., Kong, A., & Ahmed, S. K. (2016). Recurrent head and neck cancer: United Kingdom National Multidisciplinary Guidelines. J Laryngol Otol, 130(S2), S181-s190. https://doi.org/10.1017/s002221511600061x
3	Rueda, A., Giralt, J., Manos, M., Lozano, A., Sistiaga, A., Garcia-Miragall, E., Cacicedo, J., Esteban, F., Scola, B., Contreras, J., Ruiz, A., Carral, A., Sanchez-Aniceto, G., Pastor, M., Herranz, J. J., Bernal, M., & Mesia, R. (2017). Multidisciplinary management of head and neck cancer: First expert consensus using Delphi methodology from the Spanish Society for Head and Neck Cancer (part 2). Oral Oncology, 70, 65-72. https://doi.org/10.1016/j.oraloncology.2017.04.005
3	Prabhash, K., Babu, G., Chaturvedi, P., Kuriakose, M., Birur, P., Anand, A., Kaushal, A., Mahajan, A., Syiemlieh, J., Singhal, M., Gairola, M., Ramachandra, P., Goyal, S., John, S., Nayyar, R., Patil, V., Rao, V., Roshan, V., & Rath, G. K. (2020). Indian clinical practice consensus guidelines for the management of very advanced disease of squamous cell carcinoma of head and neck [Review]. Indian Journal of Cancer, 57(5), S22-S25. https://doi.org/10.4103/0019-509X.278977
3	Misiukiewicz, K., Gupta, V., & Posner, M. (2017). The Italian Collaborative Group sets a standard for the treatment of locally advanced head and neck cancer [Editorial]. Annals of Oncology, 28(9), 2051-2054. https://doi.org/10.1093/annonc/mdx394
4	Janot, F., Baglin, A. C., & Baujat, B. (2015). Management of rare head and neck cancers: Recommendations from the french network REFCOR [Article]. Oncologie, 17(5-6), 256-258. https://doi.org/10.1007/s10269-015-2523-2
6	Wierzbicka, M., & Napierała, J. (2017). Updated National Comprehensive Cancer Network guidelines for treatment of head and neck cancers 2010-2017. Otolaryngol Pol, 71(6), 1-6. https://doi.org/10.5604/01.3001.0010.7193
6	Doscher, J., & Hoffmann, T. K. (2021). Guideline for the Treatment of Patients with Head and Neck Cancer - A critical View. Laryngo-Rhino-Otologie, 100(10), 767-769. https://doi.org/10.1055/a-1623-6848

6	Prabhash, K., Babu, G., Chaturvedi, P., Kuriakose, M., Birur, P., Anand, A., Kaushal, A., Mahajan, A., Syiemlieh, J., Singhal, M., Gairola, M., Ramachandra, P., Goyal, S., John, S., Nayyar, R., Patil, V., Rao, V., Roshan, V., & Rath, G. K. (2020). Indian clinical practice consensus guidelines for the management of squamous cell carcinoma of head and neck [Review]. Indian Journal of Cancer, 57(5), S1-S5. https://doi.org/10.4103/0019-509X.278971
6	Adelstein, D. J., Ismaila, N., Ku, J. A., Burtness, B., Swiecicki, P. L., Mell, L., Beitler, J. J., Gross, N., Jones, C. U., Kaufman, M., Le, Q. T., Semrad, T. J., Siu, L. L., & Ridge, J. A. (2019). Role of Treatment Deintensification in the Management of p16+ Oropharyngeal Cancer: ASCO Provisional Clinical Opinion. J Clin Oncol, 37(18), 1578-1589. https://doi.org/10.1200/jco.19.00441
6	Bahadur, S., & Thakar, A. (2019). Guidelines in management of early laryngeal cancers (T 1 and T 2). In Carcinoma of the Larynx and Hypopharynx (pp. 63-75). https://doi.org/10.1007/978-981-13-3110-7_5
6	Wilson, J. A. (2008). Does pharma fund the pipers? - Scottish Intercollegiate Guidelines Network (SIGN) guidelines 90 - diagnosis and management of head and neck cancer, October 2006. Clin Oncol (R Coll Radiol), 20(9), 661-663. https://doi.org/10.1016/j.clon.2008.03.013
7	Society of Surgical Oncology Practice Guidelines. Laryngeal cancer surgical practice guidelines. (1997). Oncology (Williston Park), 11(8), 1201-1203, 1207-1208.
7	Society of Surgical Oncology Practice Guidelines. Oropharyngeal and oral cavity cancer surgical practice guidelines. (1997). Oncology (Williston Park), 11(8), 1211-1212, 1215-1216.
8	Forastiere, A., Goepfert, H., Goffinet, D., Hong, K. W., Laramore, G., Mittal, B., Pfister, D. G., Ridge, J., Schuller, D., Shah, J., Spencer, S., Urba, S., & Wolf, G. (1998). NCCN practice guidelines for head and neck cancer. National Comprehensive Cancer Network. Oncology (Williston Park), 12(7a), 39-147.
8	Pfister, D. G., Ang, K., Brockstein, B., Colevas, A. D., Ellenhorn, J., Goepfert, H., Hicks, W. L., Jr., Hong, W. K., Kies, M. S., Lydiatt, W., McCaffrey, T., Mittal, B. B., Ridge, J. A., Schuller, D. E., Shah, J. P., Spencer, S., Trotti, A., 3rd, Urba, S., Weymuller, E. A., Jr., . . . Wolf, G. T. (2000). NCCN Practice Guidelines for Head and Neck Cancers. Oncology (Williston Park), 14(11a), 163-194.
8	Forastiere, A. A., Ang, K., Brizel, D., Brockstein, B. E., Dunphy, F., Eisele, D. W., Goepfert, H., Hicks Jr, W. L., Kies, M. S., Lydiatt, W. M., Maghami, E., McCaffrey, T., Mittal, B. B., Pfister, D. G., Pinto, H. A., Posner, M. R., Ridge, J. A., Samant, S., Schuller, D. E., . . . Yueh, B. (2005). Head and neck cancers: Clinical practice guidelines [Review]. JNCCN Journal of the National Comprehensive Cancer Network, 3(3), 316-391. https://doi.org/10.6004/jnccn.2005.0019
8	Forastiere, A. A., Ang, K. K., Brizel, D., Brockstein, B. E., Burtness, B. A., Cmelak, A. J., Colevas, A. D., Dunphy, F., Eisele, D. W., Goepfert, H., Hicks Jr, W. L., Kies, M. S., Lydiatt, W. M., Maghami, E., Martins, R., McCaffrey, T., Mittal, B. B., Pfister, D. G., Pinto, H. A., . . . Worden, F. (2008). Head and neck cancers [Review]. JNCCN Journal of the National Comprehensive Cancer Network, 6(7), 646-695. https://doi.org/10.6004/jnccn.2008.0051
8	Zhang, B., & Tang, P. Z. (2009). [Introduction to "NCCN clinical practice guidelines in head and neck cancer (2009 Chinese version)"]. Zhonghua Er Bi Yan Hou Tou Jing Wai Ke Za Zhi, 44(9), 707-709.
8	Pfister, D. G., Ang, K. K., Brizel, D. M., Burtness, B. A., Cmelak, A. J., Colevas, A. D., Dunphy, F., Eisele, D. W., Gilbert, J., Gillison, M. L., Haddad, R. I., Haughey, B. H., Hicks Jr, W. L., Hitchcock, Y. J., Kies, M. S., Lydiatt, W. M., Maghami, E., Martins, R., McCaffrey, T., . . . Worden, F. (2011). Head and neck cancers: Clinical practice guidelines in oncology [Review]. JNCCN Journal of the National Comprehensive Cancer Network, 9(6), 596-650. https://doi.org/10.6004/jnccn.2011.0053
8	Pfister, D. G., Ang, K. K., Brizel, D. M., Burtness, B. A., Busse, P. M., Caudell, J. J., Cmelak, A. J., Colevas, A. D., Dunphy, F., Eisele, D. W., Gilbert, J., Gillison, M. L., Haddad, R. I., Haughey, B. H., Hicks Jr, W. L., Hitchcock, Y. J., Kies, M. S., Lydiatt, W. M., Maghami, E., . . . Hughes, M. (2013). Head and Neck Cancers, version 2.2013 [Article]. JNCCN Journal of the National Comprehensive Cancer Network, 11(8), 917-923. https://doi.org/10.6004/jnccn.2013.0113
8	Pfister, D. G., Spencer, S., Brizel, D. M., Burtness, B., Busse, P. M., Caudell, J. J., Cmelak, A. J., Colevas, A. D., Dunphy, F., Eisele, D. W., Gilbert, J., Gillison, M. L., Haddad, R. I., Haughey, B. H., Hicks, W. L., Jr., Hitchcock, Y. J., Jimeno, A., Kies, M. S., Lydiatt, W. M., . . . Hughes, M. (2014). Head and neck cancers, Version 2.2014. Clinical practice guidelines in oncology. J Natl Compr Canc Netw, 12(10), 1454-1487. https://doi.org/10.6004/jnccn.2014.0142
8	Pfister, D. G., Spencer, S., Brizel, D. M., Burtness, B., Busse, P. M., Caudell, J. J., Cmelak, A. J., Colevas, A. D., Dunphy, F., Eisele, D. W., Foote, R. L., Gilbert, J., Gillison, M. L., Haddad, R. I., Haughey, B. H., Hicks, W. L., Jr., Hitchcock, Y. J., Jimeno, A., Kies, M. S., . . . Hughes, M. (2015). Head and Neck Cancers, Version 1.2015. J Natl Compr Canc Netw, 13(7), 847-855; quiz 856. https://doi.org/10.6004/jnccn.2015.0102
8	Adelstein, D., Gillison, M. L., Pfister, D. G., Spencer, S., Adkins, D., Brizel, D. M., Burtness, B., Busse, P. M., Caudell, J. J., Cmelak, A. J., Colevas, A. D., Eisele, D. W., Fenton, M., Foote, R. L., Gilbert, J., Haddad, R. I., Hicks, W. L., Hitchcock, Y. J., Jimeno, A., . . . Darlow, S. D. (2017). NCCN Guidelines Insights: Head and Neck Cancers, Version 2.2017. J Natl Compr Canc Netw, 15(6), 761-770. https://doi.org/10.6004/jnccn.2017.0101
8	Colevas, A. D., Yom, S. S., Pfister, D. G., Spencer, S., Adelstein, D., Adkins, D., Brizel, D. M., Burtness, B., Busse, P. M., Caudell, J. J., Cmelak, A. J., Eisele, D. W., Fenton, M., Foote, R. L., Gilbert, J., Gillison, M. L., Haddad, R. I., Hicks, W. L., Hitchcock, Y. J., . . . Darlow, S. D. (2018). NCCN Guidelines Insights: Head and Neck Cancers, Version 1.2018. J Natl Compr Canc Netw, 16(5), 479-490. https://doi.org/10.6004/jnccn.2018.0026
8	Pfister, D. G., Spencer, S., Adelstein, D., Adkins, D., Anzai, Y., Brizel, D. M., Bruce, J. Y., Busse, P. M., Caudell, J. J., Cmelak, A. J., Colevas, A. D., Eisele, D. W., Fenton, M., Foote, R. L., Galloway, T., Gillison, M. L., Haddad, R. I., Hicks, W. L., Hitchcock, Y. J., . . . Darlow, S. D. (2020). Head and Neck Cancers, Version 2.2020, NCCN Clinical Practice Guidelines in Oncology. J Natl Compr Canc Netw, 18(7), 873-898. https://doi.org/10.6004/jnccn.2020.0031
8	Caudell, J. J., Gillison, M. L., Maghami, E., Spencer, S., Pfister, D. G., Adkins, D., Birkeland, A. C., Brizel, D. M., Busse, P. M., Cmelak, A. J., Colevas, A. D., Eisele, D. W., Galloway, T., Geiger, J. L., Haddad, R. I., Hicks, W. L., Hitchcock, Y. J., Jimeno, A., Leizman, D., . . . Darlow, S. D. (2022). NCCN Guidelines® Insights: Head and Neck Cancers, Version 1.2022. J Natl Compr Canc Netw, 20(3), 224-234. https://doi.org/10.6004/jnccn.2022.0016
8	Mesía Nin, R., Pastor Borgoñón, M., Cruz Hernández, J. J., & Isla Casado, D. (2010). SEOM clinical guidelines for the treatment of head and neck cancer. Clin Transl Oncol, 12(11), 742-748. https://doi.org/10.1007/s12094-010-0589-2
8	Mesía, R., Pastor, M., Grau, J. J., & del Barco, E. (2013). SEOM clinical guidelines for the treatment of head and neck cancer (HNC) 2013. Clin Transl Oncol, 15(12), 1018-1024. https://doi.org/10.1007/s12094-013-1096-z
8	Docampo, L. C. I., Arrula, V. A., Rotllan, N. B., Maseda, A. C., Cirauqui, B. C., Escobar, Y., Sorrosal, J. J. L., Borgonon, M. P., Rueda, A., & Hernandez, J. J. C. (2018). SEOM clinical guidelines for the treatment of head and neck cancer (2017). Clinical & Translational Oncology, 20(1), 75-83. https://doi.org/10.1007/s12094-017-1776-1
8	Quon, H., Yom, S. S., Garg, M. K., Lawson, J., McDonald, M. W., Ridge, J. A., Saba, N., Salama, J., Smith, R., Yeung, A. R., & Beitler, J. J. (2010). ACR Appropriateness Criteria: local-regional therapy for resectable oropharyngeal squamous cell carcinomas. Curr Probl Cancer, 34(3), 175-192. https://doi.org/10.1016/j.currproblcancer.2010.04.004
8	Pivot, X. (2003). ESMO Minimum Clinical Recommendations for diagnosis, treatment and follow-up of squamous cell carcinoma of the head and neck. Ann Oncol, 14(7), 1014-1015. https://doi.org/10.1093/annonc/mdg295
8	Pivot, X., Kataja, V. V., & Jelic, S. (2005). ESMO Minimum Clinical Recommendations for diagnosis, treatment and follow-up of squamous cell carcinoma of the head and neck (SCCHN). Ann Oncol, 16 Suppl 1, i62-63. https://doi.org/10.1093/annonc/mdi831
8	Pivot, X., & Villanueva, C. (2007). Squamous cell carcinoma of the head and neck: ESMO clinical recommendations for diagnosis, treatment and follow-up. Ann Oncol, 18 Suppl 2, ii65-66. https://doi.org/10.1093/annonc/mdm042
8	Pivot, X., & Felip, E. (2008). Squamous cell carcinoma of the head and neck: ESMO clinical Recommendations for diagnosis, treatment and follow-up. Ann Oncol, 19 Suppl 2, ii79-80. https://doi.org/10.1093/annonc/mdn097
8	Licitra, L., & Felip, E. (2009). Squamous cell carcinoma of the head and neck: ESMO clinical recommendations for diagnosis, treatment and follow-up. Ann Oncol, 20 Suppl 4, 121-122. https://doi.org/10.1093/annonc/mdp149
8	Grégoire, V., Lefebvre, J. L., Licitra, L., & Felip, E. (2010). Squamous cell carcinoma of the head and neck: EHNS-ESMO-ESTRO Clinical Practice Guidelines for diagnosis, treatment and follow-up. Ann Oncol, 21 Suppl 5, v184-186. https://doi.org/10.1093/annonc/mdq185
8	Pfister, D. G., Laurie, S. A., Weinstein, G. S., Mendenhall, W. M., Adelstein, D. J., Ang, K. K., Clayman, G. L., Fisher, S. G., Forastiere, A. A., Harrison, L. B., Lefebvre, J. L., Leupold, N., List, M. A., O'Malley, B. O., Patel, S., Posner, M. R., Schwartz, M. A., & Wolf, G. T. (2006). American Society of Clinical Oncology clinical practice guideline for the use of larynx-preservation strategies in the treatment of laryngeal cancer. J Clin Oncol, 24(22), 3693-3704. https://doi.org/10.1200/jco.2006.07.4559

*(1) CPGs without treatment recommendations; (2) CPGs focused entirely on unique techniques; (3) CPGs for treatment of HNC recurrences and metastases; (4) CPGs on HNC topographies other than oral cavity, oropharynx, and larynx; (5) non-SCC CPGs; (6) non-CPGs study designs; (7) full texts not available; and (8) outdated versions of CPGs published by the same societies.

Table S3. Treatment recommendation for oral cavity per society according to clinical stages

Country	Society	Early stage (I-II) oral cancer	Advanced stage (III-IV) oral cancer
Africa	AHNS	-Surgery<br>-NT: observation (For T1N0M0 with a depth of invasion 3 mm or less) or elective neck dissection (Level of evidence: 1)	-Surgery + reconstruction<br>-NT: radical neck dissection<br>-If adjuvant therapy is not available, a cuff of 1.5 cm of normal surrounding tissue should be resected (Level of evidence: 2B)<br>-If surgery alone is unable and adjuvant CRT is not available, palliative care should be considered (Level of evidence: 2B)
Asia	APHNCEP	-Surgery (preferred) or RT (external-beam, brachytherapy)<br>-NT: Ipsilateral or bilateral neck dissection	-Surgery or CCRT (tumors unsuitable for surgery or with unresectable disease) or RT alone<br>-NT: neck dissection or observation in N0 neck disease after CCRT
Asia	ESMO-KSMO	-Surgery followed by postoperative RT or CRT if indicated [IV,A] or RT [IV,B] (brachytherapy for primary (selected T1) [III,B]	-Surgery followed by postoperative RT or CRT if indicated [IV,A] or definitive CRT (contraindications to surgery, including functional unresectability) [IV,B]<br>-cT4b cM0 and/or unresectable lymph nodes: CCRT [III or IV,B] or ICT followed by RT or CRT for responders [IV,B], or palliative treatment
China	CSCO	-Surgery or RT (only considered for patients who do not meet the criteria for surgery)	-Surgery (preferred) + reconstruction and postoperative adjuvant RT<br>-NT: selective or radical neck dissection<br>-No criteria for surgery: CCRT (cisplatin) or RT alone (for patients who are not suitable for cisplatin) or sequential therapy with ICT combined with RT
Denmark	DSHNO	-Surgery or RT<br>-NT: elective neck dissection	-Surgery and postoperative RT<br>-NT: neck dissection
Europe	EHNS-ESMO-ESTRO	-Surgery followed by postoperative RT or CRT if indicated [IV,A] or RT [IV,B] (brachytherapy for primary (selected T1) [III,B])	-Surgery followed by postoperative RT or CRT if indicated [IV,A] or definitive CRT (contraindications to surgery) [IV,B]<br>-cT4b cM0 and/or unresectable lymph nodes: CCRT or ICT followed by RT or CRT for responders, or palliative treatment [III or IV,B]
India	OCTF-MEP	-Surgery (preferred) or definitive RT [IA]<br>-NT: elective neck dissection (preferred) or SLN biopsy	-Surgery (preferred) followed by RT/CTRT<br>-NT: ipsilateral, or bilateral neck dissection<br>-Patients unfit for surgery: neoadjuvant ChT (Responders may go for surgery followed by CTRT OR RT OR palliative treatment)
India	ICMR	-Surgery (preferred) or RT<br>-NT: observe (in clinically node negative) or treat electively	-Surgery followed by postoperative RT or CRT<br>-NT: neck dissection<br>-Locally advanced inoperable cancers (stage IVB): palliative CRT, ChT, RT or symptomatic treatment according to performance status<br>-Select cases: neoadjuvant ChT followed by surgical salvage
Japan	JSHNC	-Surgery (preferred) or RT (brachytherapy) (Grade C1)<br>-NT: elective or prophylactic neck dissection (Grade C1)	-ChT (Platinum-based ChT is performed as induction ChT for advanced cancer) (Grade C1).
Spain	SSHNC	-Surgery (preferred) or RT	-Surgery (resectable III-IVA with subsequent reconstruction expected) or CRT<br>-Stage III-IVb who undergo a non-surgical treatment approach: IMRT (preferred modality) or CCRT<br>-IVA-B N2b-c/N3: CCRT or ICT (No recommended in unfit patients (performance status)
Spain	SEOM	-Transoral resection (preferred) or RT (II, B)<br>-NT: prophylactic RT or elective neck dissection (II, B) or SLN biopsy for T1–2N0 (I, A)	-Surgery (preferred) or CCRT (no criteria for surgery) (IA) or ICT followed CCRT/RT alone (IIB) or RT alone (I-A) (not candidates for platinum)<br>-NT: T3-4aN0: ipsilateral or bilateral neck dissection is mandatory (I-A).
UK	NICE	-NT: neck dissection or SLN biopsy (T1–T2, N0)	
UK	UKNMG	-Surgery (preferred) + reconstruction or brachytherapy (selected cases)<br>-NT: SLN biopsy or elective neck dissection	-Surgery + reconstruction and post-operative RT<br>-NT: neck dissection
USA	CPG for HNC	-Surgery (preferred)<br>-Postoperative RT is recommended for selected patients	-Surgery + reconstruction (preferred)<br>-All procedures require postoperative RT<br>-NT: neck dissection

USA	NCCN	-Surgery (preferred) or definitive RT<br>-NT: Neck dissection or SLN biopsy	-Surgery (preferred) or clinical trials<br>-No criteria for surgery: CCRT or definitive RT (if unfit for CCRT)<br>-NT: ipsilateral or bilateral neck dissection

Abbreviations: AHNS, African Head and Neck Society; APHNCEP, Asia Pacific HNC Expert Panel; CSCO, Chinese Society of Clinical Oncology; CPG, clinical practice guideline; DSHNO, Danish Society for Head and Neck Oncology; EHNS, European Head and Neck Society; ESMO, European Society for Medical Oncology; ESTRO, European Society for Radiotherapy and Oncology; HNC, head and neck cancer; ICMR, Indian Council of Medical Research; JSHNC, Japan Society for Head and Neck Cancer; KSMO, Korean Society of Medical Oncology; NCCN, National Comprehensive Cancer Network; NICE, National Institute for Health and Care Excellence; NT, neck treatment; OCTF-MEP, Oral Cancer Task Force with a Multidisciplinary Expert Panel; SSHNC, Spanish Society for Head and Neck Cancer; SEOM, Spanish Society of Medical Oncology; UKNMG United Kingdom National Multidisciplinary Guidelines;

Table S4. Treatment recommendation for oropharyngeal cancer per society according to clinical stages

Country	Guidelines	Early stage (I-II) oropharyngeal cancer	Advanced stage (III-IV) oropharyngeal cancer
Asia	APHNCEP	-RT (preferred) or surgery (selected cases)	-CRT (cisplatin or cetuximab) or RT alone<br>-NT: neck dissection (T3N2), in addition to RT plus systemic therapy
Asia	ESMO-KSMO	-p16 (-) or (+): RT or transoral surgery followed by RT or CRT if indicated [IV,A]	-p16 (-) or (+): CCRT [IV,A] or surgery followed by RT or CRT if indicated [IV,B]
China	CSCO	-Surgery or RT (no criteria for surgery) (class 2A evidence)<br>-NT: ipsilateral or bilateral selective neck dissection	-T1−2, N1−2: surgery followed by postoperative RT/CRT or CCRT (if functional protection is required).<br>-T3−4: CCRT (preferred to preserve functions) (class 1A evidence) or RT alone (elderly patients)<br>-T4 or N2c−N3: ICT (may be considered to reduce tumor mass and reduce the risk of metastasis) (class 1B evidence)
Europe	EHNS-ESMO-ESTRO	-p16 (-) or (+): RT or transoral surgery followed by RT or CRT if indicated [IV,A]	-p16 (-) or (+): CCRT [IV,A] or surgery followed by RT or CRT if indicated [IV,B]
India	OCTF-MEP	-p16 (-) T1–2,N0–1 / p16 (+)T1–2,N0: Transoral/open surgery or definitive RT (no criteria for surgery) [IA]<br>-p16 (-) (T1–2,N1 only)/p16(+) (T2 only): CCRT [IA]<br>-NT: ipsilateral or bilateral neck dissection (preferred)	-T3–4a,N0–1; T1–4a,N2–3, p16 (-)/(+): CCRT + Nimotuzumab [IA] or resection of primary [IIIB] or ICT followed by RT/CRT<br>-NT: ipsilateral or bilateral neck dissection
Japan	JSHNC	-Transoral resection<br>-NT: prophylactic neck dissection (level II–III or II–IV)	-CRT or ICT<br>-NT: When control of cervical metastasis is considered to be difficult by CCRT, neck dissection may be performed prior to CRT
Spain	SSHNC	-p16 (+) I/II: RT (preferred over surgery) (although there is no consensus)<br>-p16 (-) I/II: surgery (preferred over RT) (although there is no consensus)	-CCRT or ICT (cetuximab is preferred over continuance with cisplatin-based CRT)<br>-CCRT and ICT are not recommended in unfit patients (poor performance status)
Spain	SEOM	-TORS/TLM (preferred) or open surgery or RT (II, B)<br>-HPV-related OPC: TORS or TLM or IMRT (IA).<br>-NT: prophylactic RT or elective neck dissection (II, B) or SLN biopsy for T1–2N0 (I, A)	-Surgery (preferred) or CCRT (no criteria for surgery) (IA) or ICT followed by CCRT/RT alone (IIB) or RT alone (I-A)<br>-HPV-related oropharyngeal cancer: clinical trials (deintensification protocols should be undertaken only within the context of clinical trials)<br>-NT: ipsilateral or bilateral neck dissection (I-A). If neck nodes are palpable, all nodal levels should be dissected
UK	UKNMG	-T1–T2 N0: surgery (TLM or TORS) or RT<br>-NT: elective neck dissection or RT	-T3-4,N0 and T1-4, N1-3: Primary CRT (IMRT preferred) or transoral surgery and adjuvant CRT<br>-Postoperative CRT or postoperative RT alone (patients who have adverse features after surgery)<br>-NT: RT or selective neck dissection<br>-N2 or N3 disease receiving radical CRT should have a PET-CT scan, with a subsequent neck dissection if residual nodal is detected (R)
UK	NICE	-Transoral surgical resection or RT<br>-Consider postoperative RT or CCRT (if pathologic adverse risk factors)	
USA	ACR-AC	-T1-T2,N0: surgery or RT<br>-T1-2,N1-2a: RT or CRT or transoral surgery<br>-T1-2N2b-3: CRT or transoral surgery<br>-NT: neck dissection	-T3-T4,N0-N2a: CCRT (preferred)<br>-T1-T2,N2b-N3 either HPV (+) or (-): CCRT or transoral surgery + neck dissection and adjuvant therapy<br>-T3-T4,N2b-N3: CCRT (external radiation and cisplatin)<br>-NT: neck dissection

USA	CPG for HNC	-Surgery or RT<br>-NT: neck dissection<br>-Postoperative RT (if adverse features)	-Surgical excision + reconstruction and postoperative RT
USA	NCCN	-p16 (-) T1–2,N0–1: surgery or definitive RT<br>-T1–2,N1 only: CCRT (category 2B) or clinical trials<br>-p16 (+)T0–2,N0: surgery or definitive RT or clinical trials<br>-p16 (+) T0–2,N1 (single node ≤3 cm): surgery or definitive RT or CCRT (category 2B) or clinical trials<br>-NT: ipsilateral or bilateral neck dissection	-p16 (-) T3–4a,N0–1; T1–4a,N2–3 / p16 (+) T0–2,N1; T0–2,N2 or T3,N0–2: CCRT or surgery or ICT (category 3) followed by RT or CCRT or clinical trials<br>-p16 (+) T0–3,N3 or T4,N0–3: CCRT (preferred) or surgery or ICT (category 3) followed by RT or CCRT or clinical trials<br>-NT: ipsilateral or bilateral neck dissection

(-) negative (+) positive; abbreviations: ACR-AC, American College of Radiology-Appropriateness Criteria; APHNCEP, Asia Pacific HNC Expert Panel; CSCO, Chinese Society of Clinical Oncology; CPG, clinical practice guideline; EHNS, European Head and Neck Society; ESMO, European Society for Medical Oncology; ESTRO, European Society for Radiotherapy and Oncology; HNC, head and neck cancer; ICMR, Indian Council of Medical Research; JSHNC, Japan Society for Head and Neck Cancer; KSMO, Korean Society of Medical Oncology; NCCN, National Comprehensive Cancer Network; NICE, National Institute for Health and Care Excellence; NT, neck treatment; OCTF-MEP, Oral Cancer Task Force with a Multidisciplinary Expert Panel; SSHNC, Spanish Society for Head and Neck Cancer; SEOM, Spanish Society of Medical Oncology; UKNMG United Kingdom National Multidisciplinary Guidelines; CRT: concurrent chemoradiotherapy; CRT, chemoradiotherapy; ICT, induction chemotherapy; OPC, oropharyngeal cancer.

Table S5. Treatment recommendation for larynx cancer per society according to clinical stages

Country	Guidelines	Early stage (I-II) laryngeal cancer	Advanced stage (III-IV) laryngeal cancer
Asia	APHNCEP	-Glottic: RT or endoscopic surgery with carbon dioxide laser resection<br>-Supraglottic: radical RT is preferred over endoscopic or open partial laryngectomy	-CCRT or RT alone as the preferred options.<br>-Patients considered for a surgical approach: partial laryngectomy or total laryngectomy with the possibility of ipsilateral thyroidectomy with or without neck dissection in case of nodal involvement.<br>-Patients having non-functional larynx or cartilage destruction, should be considered for upfront a surgical treatment, laryngectomy or laryngopharyngectomy, followed by adjuvant RT or CCRT, depending on the risk of recurrence.<br>-For alternative organ preservation strategy: taxane-platinum-5-FU (TPF) regimen, over PF, followed by RT in highly selected patients.
Canada	CCOHNCDG	-Endolaryngeal surgery (with or without laser) or RT	
China	CSCO	-Surgery (TLS or TORS if possible) or RT alone (class 2A evidence) (if no criteria for surgery)	-Total laryngectomy followed by postoperative RT or CRT<br>-For patients who desire to preserve the throat function: CRT or ICT followed by RT (If patients reach complete or partial remission after ICT)<br>-Patients >70 years old who have unclear survival benefits with concurrent treatment, RT alone may be used<br>-NT: Selective or radical bilateral node dissection
Europe	EHNS-ESMO-ESTRO	-cT1-3 cN0-3 cM0: Surgery (laser) followed by RT or CRT if indicated [IV,A]<br>-T1-2, N0: RT [IV,A]<br>-T3 or N1-3: CRT [IV,A]	-cT1-2 cN2-3, cT3b cN0-3: CCRT (T and N) [I,A]<br>-If total laryngectomy is necessary: CCRT or ICT followed by RT (in case of complete or partial response after induction or surgery) [I,A]<br>-Option: Surgery and lymph node dissection followed by RT or CRT if indicated [IV,B]<br>-cT4a cN0-3: Surgery followed by RT or CRT [IV,A]<br>-Option: CCRT [IV,B]<br>-cT4b cN0-3: ICT followed by RT or CCRT [IV,B] or palliative treatment.
Germany	DKG-DKH	-cT1 cN0 and cT2 cN0: TLM or RT<br>-NT: elective or therapeutic neck dissection<br>-NT: cT1 cN0 glottic carcinomas should not receive an elective neck dissection	-Surgery + adjuvant RT/CRT or primary CRT (possibly followed by salvage surgery) or ICT+ surgery/RT/CRT or EGF receptor inhibitors combined with RT/CRT<br>-Laryngeal organ preservation (cT1-2 N+, cT3-4): Primary resection followed by adjuvant RT/CRT or by primary CRT.<br>-If a surgical laryngectomy is necessary: ICT or chemoantibody therapy with subsequent RT or CRT or subsequent resection (if the response to ICT is poor)<br>-If no criteria for resection: primary CRT should be performed.

			-NT: unilateral or bilateral elective or therapeutic neck dissection.<br>-After primary RT or CRT, neck dissection should only be performed if imaging lymph nodes (+)
India	OCTF-MEP	-in situ; T1–2, N0: TLM (preferred) or RT<br>-T1-2,N0: partial laryngectomy/endoscopic or open resection<br>-NT: neck dissection or definitive RT [1A]	-Organ preservation: CCRT or ICT with TPF (docetaxel, cisplatin, and FU) followed by definitive RT or definitive CCRT<br>-Patients who are not fit for laryngeal preservation: surgery (laryngectomy with thyroidectomy, ipsilateral or bilateral neck dissection, and pretracheal and ipsilateral paratracheal lymph node dissection) with RT or CTRT.<br>-Glottic and supraglottic T4a, N0–3: total laryngectomy with thyroidectomy ± neck dissection and paratracheal lymph node dissection, as indicated, followed by adjuvant treatment.
Japan	JSHNC	-RT or surgery (Grade A).<br>-NT: RT (except cervical lymph node in glottic cancers)<br>-For recurrences following RT in early-stage glottic cancer, larynx-preserving surgery is often performed as salvage surgery (Grade B).	-CCRT (Grade B) or ICT (Grade C1)<br>-For T4 cases with invasion into the soft tissue over thyroid cartilage: total laryngectomy<br>-NT: neck dissection. In progressive subglottic cancers, ipsilateral lobes of the thyroid and paratracheal lymph nodes are dissected at the time of total laryngectomy.
Asia	Pa ESMO-KSMO	-cT1-3 cN0-3 cM0: Surgery (laser) followed by RT or CRT if indicated [IV,A]<br>-T1-2, N0: RT [IV,A]<br>-T3 or N1-3: CRT [IV,A]	-cT1-2 cN2-3, cT3b cN0-3: CCRT (T and N) [I,A]<br>-If total laryngectomy is necessary: CCRT or ICT followed by RT (in case of complete or partial response after induction or surgery) [I,A]<br>-Option: Surgery and lymph node dissection followed by RT or CRT if indicated [IV,B]<br>-cT4a cN0-3: Surgery followed by RT or CRT [IV,A]<br>-Option: CCRT [IV,B]<br>-cT4b cN0-3: ICT followed by RT or CCRT [IV,B] or palliative treatment.
Netherlands	DCHNCG	-Carcinoma in situ: Endoscopic decortication (laser preferred) (level 2) or RT (If surgery is not feasible because of the extension of the lesion or technical limitations)<br>-T1 glottic: RT (level 3) or Endoscopic decortication (an alternative for superficial T1 lesions)<br>-T1 supraglottic carcinoma: RT (level 3)<br>-NT: Elective neck treatment should not be given to patients with T1 or T2 glottic carcinoma with normal mobility of the cord or T1 supraglottic carcinoma (level 3).	-T2–T3–T4: A function preserving treatment should be the first choice for every patient with a larynx carcinoma (level 3)<br>-T2 supraglottic tumors and for T3 and T4 glottic/supraglottic tumors: RT (level 1) or supraglottic laryngectomy or endoscopic partial laryngectomy (selected cases) (level 3).<br>-NT: Elective neck dissection (level 3). In tumors with subglottic extension, the paratracheal nodes should be treated electively (level 3).<br>-For nodal metastases smaller than 3 cm: RT alone; larger than 3 cm: neck dissection with postoperative RT (level 3).
Spain	SSHNC	-Surgery (preferred) or RT (treatment of choice in patients who need to preserve their voice for professional purposes).	-Supraglottic laryngectomy or CCRT<br>-T3: CCRT or total/partial laryngectomy<br>-T4: surgery followed by adjuvant RT/CRT should be the treatment of choice.<br>-The treatment of choice when avoiding a pharyngolaryngectomy should be: CCRT or ICT
Spain	SEOM	-Conservative surgery (preferred) (TLM) or open surgery or RT<br>-NT: elective treatment of supraglottic cancer is recommended (II, B), but not in glottic cancer (III, C)	-Surgery (preferred) or CCRT (patients that are not candidates or refuse radical surgery) (IA) or ICT followed by CCRT or RT alone (IIB) or RT alone (I-A) (Unfit patients not candidate for platinum)<br>-Organ preservation:<br>-Surgical resection (total versus partial laryngectomy + neck dissection) followed by RT (IA) (Specially in T4a and for the most part of subglottic tumors (IA))<br>-CRT (if patient refuses surgery) (IA) (Consider cetuximab if cisplatin cannot be administered) (IA).<br>-ICT with TPF schedule
UK	UKNMG	-Glottic: RT or TLM or open partial surgery (selected tumors) (R)<br>-NT glottic: elective neck dissection is not recommended (low risk of occult nodal disease)<br>-Supraglottic: RT or TLM or TORS (R) or supraglottic laryngectomy (selected tumors) (R)<br>-NT supraglottic: elective treatment of at least bilateral lymph node levels II and III, either with RT or selective neck dissection	-T2b–T3 glottic, T3 supraglottic, non-surgical larynx preservation therapies: CCRT (preferred) or TLM or open partial surgical procedures ± post-operative RT<br>-N2-3 with complete clinical and radiologic imaging response after CRT do not require planned neck dissection.<br>-T4 (larynx preservation): CCRT (unless there is tumour invasion through cartilage into the soft tissues of the neck) or total laryngectomy (R)<br>-NT: elective treatment (RT or surgery ± post-operative RT) is recommended

UK	NICE	-T1 and T2: TLM<br>-T1b–T2 glottic: TLM or RT<br>-T1–T2 supraglottic: Transoral surgery or RT	-T3: CCRT or surgery with adjuvant RT, with or without CCRT<br>-T4a: Surgery with adjuvant RT, with or without CCRT
USA	ASCO	-Endoscopic resection or RT<br>-NT: elective neck dissection (patients with advanced lesions of the glottis and all patients with supraglottic lesions)	-T3, T4 Organ-preservation: surgery or CCRT or RT alone<br>-Selected patients with extensive T3 or large T4a: total laryngectomy.<br>-NT: neck dissection (patients who are treated with surgery for the primary)<br>-N(+) with complete clinical and radiologic imaging response after ChT do not require elective neck dissection.
USA	ACR-AC	-Transoral endolaryngeal resection (preferred) or external beam RT or open partial laryngectomy	
USA	CPG for HNC	-RT (preferred) or surgery	-Surgery + postoperative RT and ICT followed by RT<br>-T4: total laryngectomy and postoperative RT.
USA	NCCN	-Carcinoma in situ: endoscopic resection (preferred) or RT<br>-Glottic (T1–T2,N0 or select T3,N0): RT or partial laryngectomy/endoscopic or open resection as indicated<br>-Supraglottic (Most T1–2,N0; Selected T3): endoscopic resection or open partial supraglottic laryngectomy or definitive RT<br>-NT: neck dissection (glottic cancer if indicated)	-Glottic (T3 N0–1), supraglottic (T3,N0): CCRT or RT or surgery or ICT or clinical trials<br>-NT: including ipsilateral or bilateral neck dissection; consider thyroidectomy to clear central compartment nodes (glottic). Thyroidectomy and with ipsilateral, central, or bilateral neck dissection (supraglottic)<br>-Glottic (T3 N2–3): CCRT or laryngectomy or ICT or clinical trials<br>-NT: thyroidectomy as indicated, ipsilateral or bilateral neck dissection, and pretracheal and ipsilateral paratracheal lymph node dissection<br>-Glottic (T4a,N0–3): surgery + ipsilateral or bilateral neck dissection; thyroidectomy to clear central compartment nodes<br>-Selected T4a patients who decline surgery: CCRT or clinical trial for function-preserving surgical or RT alone or ICT<br>-Supraglottic (T1–2,N+ and selected T3,N1): CCRT or definitive RT for low-volume disease or partial supraglottic laryngectomy and neck dissection(s) or ICT or clinical trials<br>-Supraglottic (most T3,N1–N3): CCRT or laryngectomy + ipsilateral thyroidectomy with neck dissection or ICT or clinical trials<br>-Supraglottic (T4a,N0–N3): laryngectomy, thyroidectomy as indicated with ipsilateral or bilateral neck dissection or CCRT or clinical trial or ICT (patients who decline surgery)

Abbreviations: ACR-AC, American College of Radiology-Appropriateness Criteria; APHNCEP, Asia Pacific HNC Expert Panel; CSCO, Chinese Society of Clinical Oncology; CPG, clinical practice guideline; EHNS, European Head and Neck Society; ESMO, European Society for Medical Oncology; ESTRO, European Society for Radiotherapy and Oncology; HNC, head and neck cancer; ICMR, Indian Council of Medical Research; JSHNC, Japan Society for Head and Neck Cancer; KSMO, Korean Society of Medical Oncology; NCCN, National Comprehensive Cancer Network; NICE, National Institute for Health and Care Excellence; NT, neck treatment; OCTF-MEP, Oral Cancer Task Force with a Multidisciplinary Expert Panel; SSHNC, Spanish Society for Head and Neck Cancer; SEOM, Spanish Society of Medical Oncology; UKNMG United Kingdom National Multidisciplinary Guidelines; CRT: concurrent chemoradiotherapy; CRT, chemoradiotherapy; ICT, induction chemotherapy; OPC, oropharyngeal cancer, DKG-DKH German Cancer Society and German Cancer Aid
